# Agreement Between Resting Energy Expenditure Predictive Formulas and Indirect Calorimetry in Non-Dialysis Dependent Chronic Kidney Disease

**DOI:** 10.3390/diagnostics14222603

**Published:** 2024-11-20

**Authors:** Mariana Cassani de Oliveira, Marina Nogueira Berbel Bufarah, Rodrigo Bueno de Oliveira, Cassiana Regina de Góes, André Luís Balbi

**Affiliations:** 1 Division of Nephrology, Department of Internal Medicine, School of Medical Sciences, University of Campinas (UNICAMP), Campinas 13060-904, SP, Brazil; rbo@unicamp.br; 2 Botucatu Medical School, Universidade Estadual Paulista (UNESP), Botucatu 14800-060, SP, Brazil; marina.berbel@unesp.br (M.N.B.B.); abalbi@unesp.br (A.L.B.); 3 Nutrition Department, Universidade Federal de Viçosa, Viçosa 36570-900, MG, Brazil; cassiana.goes@yahoo.com.br

**Keywords:** resting energy expenditure, predictive formulas, chronic kidney disease

## Abstract

Background and Aims: The gold standard method for measuring resting energy expenditure (REE) is indirect calorimetry (IC) using an expensive device that requires specialized training. To overcome the limitations of IC, REE prediction formulas are used in patients with chronic kidney disease (CKD). However, it is still controversial which of these formulas has greater accuracy compared to IC. We aimed to determine the accuracies of REE measured by IC and estimated by formulas in patients with CKD. Methods: Fifty-three patients with stage 4–5 CKD underwent IC and five current REE prediction formulas. Accuracy was measured by Lin’s correlation coefficient. Bland–Altman repeated measures analysis was used to assess the agreement of the formulas’ results with those of IC. Precision was measured by the predicted IC ± 10% and 20%. Systematic bias was assessed by the Student’s t-test, and linear regression was used to assess proportionality bias. Results: Patients had a mean estimated glomerular filtration rate (eGFR) of 12 ± 4 mL/min/1.73 m^2^, a mean age of 65 years, and 62% were male. The mean REE measured by IC was 1341 ± 37 Kcal/day, and the formula with the lowest mean bias (0.1509 [−653.5121; 398.9056]), best correlation (r = 0.789; *p* = 0.000), and best accuracy (85%) was the formula developed by Fernandes and Cols (REE (kcal/day) = 854 + (7.4 × body weight) + (179 × sex) − (3.3 × age) + (2.1 × eGFR) + 26 (if diabetes)). Conclusions: The Fernandes and Cols equation had good accuracy and was valuable for estimating energy requirements in the population studied.

## 1. Introduction

Protein-energy wasting (PEW) is a common condition in chronic kidney disease (CKD) and has a negative impact on patient prognosis, clinical management, health economics, quality of life, and morbidity [1,2]. PEW has a lower prevalence in the early stages of CKD, but is present in between 11% and 54% of patients with stages 3–5 CKD [2], and can be caused by decreased appetite and poor energy and protein intake, persistent inflammation, endocrine changes, increased circulation of uremic toxins, and the physiological aging process itself [1,3].

One of the changes common to all of these causes is a change in resting energy expenditure (REE). The mechanism of REE change in CKD is not fully understood, but it is strongly influenced by the amount of lean mass, dialysis treatment, and endocrine–metabolic diseases such as hyperparathyroidism and diabetes [4,5,6,7].

The gold standard method for measuring REE is indirect calorimetry (IC), which is highly accurate, with errors of less than 1% and high reproducibility [8]. However, IC instruments are expensive and require special training to use, making IC an impractical method for measuring REE in routine clinical practice. Several predictive formulas for REE have been developed over the years, such as the Harris–Benedict (1919) [9] and Schofield (1995) [10] methods, for populations with different clinical characteristics. Previous studies have already shown that the use of these formulas to estimate the REE of CKD patients, both on dialysis and on conservative management, is inadequate because they overestimate or underestimate the true REE of the CKD population [7,11,12]. For example, our group previously published that the Harris–Benedict formula presents poor agreement (~25%) with REE values obtained by IC due to the limits of agreement of what was considered clinically acceptable (±200 Kcal) or due to the low precision (49%) of the estimated measurement [12].

Recently, five new prediction formulas have been developed specifically for the CKD population. Vilar et al. (2014) [11] and Byham-Gray et al. (2017) [13] developed formulas using data only from individuals treated with hemodialysis. In 2019, Fernandes et al. [14] developed a formula for the population treated with both hemodialysis and peritoneal dialysis, and in 2021, the same group of researchers developed a formula for the non-dialysis CKD population [15].

Accurate determination of REE in CKD patients is extremely important for appropriate prescription of daily energy intake and consequent reduction in the risk of PEW. Therefore, the present study compared the REE of non-dialysis CKD patients measured by IC with REE calculated by predictive formulas and determined their accuracies.

## 2. Materials and Methods

### 2.1. Design and Patient Selection

This was a clinical prospective cohort study that included patients with advanced CKD from the Pre-Dialysis Outpatient Clinic of the Botucatu Medical School (São Paulo State, Brazil). This research was approved by the Ethics Committee (CAAE: 66148917.1.0000.5411) and all patients signed an informed consent form.

Non-dialysis stage 4–5 CKD patients (eGFR < 20 mL/min/1.72 m^2^) [16] older than 18 years and of both sexes were enrolled by convenience sampling. Pregnant women, patients with cancer, or patients with reduced life expectancy were excluded. Study follow-up was interrupted if the patient experienced any clinical event that could interfere with REE and/or dietary intake, such as hospitalization, surgical intervention, cardiovascular events, antibiotic therapy, or initiation of dialysis therapy, before completion of assessments. Recruitment, all IC interventions, and REE estimation using predictive formulas were performed from March 2019 to February 2020.

### 2.2. Anthropometric Measures

To measure body weight, each subject stood in the center of the base of an electronic scale (model W 200A, WELMY, São Paulo, Brazil) wearing light clothing at all measurement moments. Height was measured with a stadiometer connected to the scale. Each person stood barefoot, with their weight evenly distributed between their feet, arms extended along their body, and heels together, touching the vertical stem of the stadiometer.

### 2.3. Resting Energy Expenditure Assessment

REE measurements were performed with an IC instrument (Quark RMR, Cosmed, Rome, Italy). It was calibrated before each measurement according to the manufacturer’s instructions. Participants wore light clothing when their body weight was measured with anthropometric medical scales just before the REE measurement. Patients were instructed to take their regular medications, not to exercise for 24 h, and to sleep for eight hours before the test. They were examined in the morning after a 12 h fast. After 30 min of rest in a reclined position, subjects breathed for 20 min in a canopy, in silence, in a room with a neutral temperature (24 °C). They were instructed to avoid hyperventilation, sudden movements, or falling asleep during the test [12].

For measurements of oxygen consumption and carbon dioxide production, the first five minutes of the test were ignored and the average of the last 15 min was used. REE was calculated according to Weir’s equation [17] (Table 1) without the use of urinary urea nitrogen. The respiratory quotient (RQ) was calculated as the ratio of the volume of carbon dioxide expired to the volume of oxygen consumed. Measurements with an RQ between 0.67 and 1.30 were used to increase the reliability of values measured by IC [18].

None of the participants were prevented from taking their medication. Serum C-reactive protein (CRP) and serum creatinine were also collected after a 12 h overnight fast on the same day as the IC test. Serum creatinine was determined by a standard autoanalyzer and CRP by the technique of chemiluminescent immunoassay, both analyzed in the laboratory of the Clinical Hospital of Botucatu.

Predictive formulas were calculated and compared with the results of REE by IC. Table 1 shows the complete factors and covariates used in each formula used in this study.

### 2.4. Statistical Analysis

Continuous variables were described as means and standard deviations or medians with interquartile ranges according to the normality of the variables using the Kolmogorov–Smirnov test.

Agreements of the formulas were evaluated using Lin’s correlation coefficient of agreement (ρc). The ρc assesses the precision and accuracy of the relationship between two measurement methods and is the product of the correlation coefficient (ρ) between the paired measurements and a bias correction factor (Cb), which measures how much the line of best fit between them deviates from the 45° line. When the correlation coefficient of agreement equals one, there is perfect agreement between two variables [19,20].

Bland–Altman repeated measures analysis was used to evaluate the REE agreement between the formulas and the IC. The bias measure represented the difference between the formulas’ results and the IC measurements. A bias of zero represented perfect agreement between the methods. Two standard deviations were used to represent the limits of agreement. Systematic bias was assessed by the Student’s T-test with mean differences equal to zero. Proportionality bias was assessed by linear regression to determine if the differences were affected by the magnitude of the measurement, using the difference between the values of the dependent variable and the mean of the differences as the independent variable [19]. Accuracy was also determined and discussed using ±10% measured and ±20% predicted REE. Analyses were performed using the MedCalc statistical program (version 22.021).

## 3. Results

**Figure 1:**
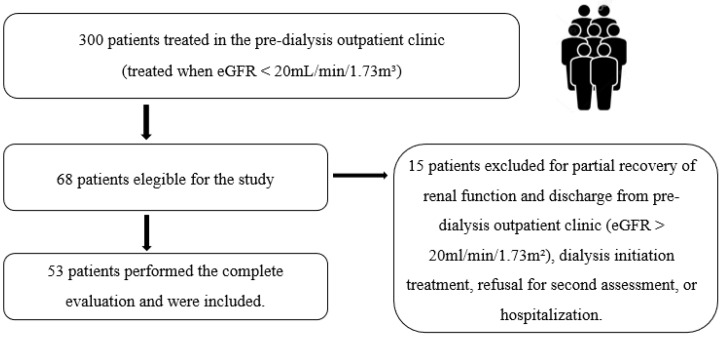
Flow chart of enrollment of 53 patients from March 2019 to February 2020.

Sixty-eight patients were eligible for this study, and fifteen were excluded due to partial recovery of renal function and discharge from the pre-dialysis outpatient clinic (eGFR > 20 mL/min/1.73 m^2^), initiation of dialysis treatment, refusal of the second assessment, or hospitalization, as shown in Figure 1. Each patient underwent three IC measurements, resulting in 159 measurements. All 53 included patients were assessed by IC and had REE estimated using the five most recent REE prediction formulas developed in the CKD population [11,13,14,15].

Most individuals were male (62%), with diabetes (38%) and hypertension (38%) being the most common underlying CKD conditions. Other demographic, nutritional, and clinical variables of these individuals are described in Table 2.

Table 3 shows the means of the three REE measures from IC tests, Bland–Altman analyses, and precision analyses at ±10% and ±20% for each formula. Mean REEs estimated by the formulas were significantly different from means generated by the IC. The formula with the lowest mean bias (0.15 [−653.51; 398.90]), the best correlation (r = 0.789; *p* = 0.0001), and the best accuracy (85.1%) was the formula developed by Fernandes et al. (2021) [15].

**Figure 2:**
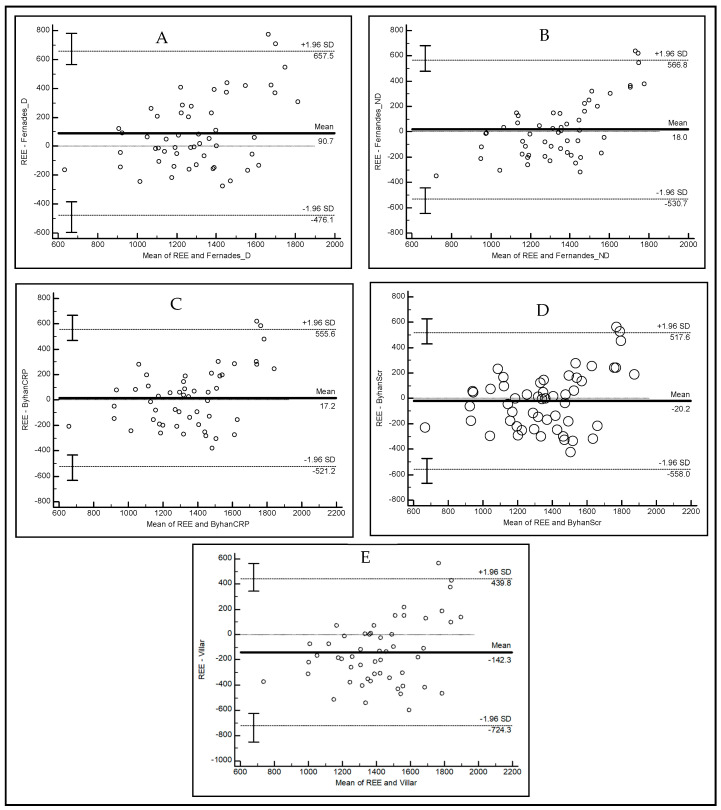
(**A**–**E**) Bland–Altman Analyses. REE: resting energy expenditure. On the *y*-axes of Figure 1 are plotted the values of differences between the two methods, and on the *x*-axes, the averages of the results of the methods. The limits of agreement were established by the mean values of the differences ± 1.96 SD. Midlines correspond to biases (means of differences between methods). (**A**) Differences between REE measured by IC and estimated by the formula of Fernandes et al. (2019) [14]; (**B**) differences between REE measured by IC and estimated by the formula of Fernandes et al. (2021) [15]; (**C**) differences between REE measured by IC and estimated by the formula of Byham-Gray et al. (2017) [13] (MHDE-CRP); (**D**) differences between REE measured by IC and estimated by the formula of Byham-Gray et al. (2017) [13] (MHDE-SCr); sizes of spheres indicate variation in repeated measurements; and (**E**) differences between REE measured by IC and estimated by the formula of Villar et al. (2014) [11].

Figure 2A–E show Bland–Altman plots for each formula tested. These plots demonstrate the presence of proportionality bias (*p* < 0.0001), which was maintained even after adjustment for log. The presence of bias indicates that the difference between the methods (REE measured by IC and estimated by predictive equations) was influenced by the magnitude of the measurements; i.e., differences between methods were greater for REE measurements that were far above or below the REE mean. This was expected because these predictive equations do not consider all variables that influence or explain energy expenditure.

## 4. Discussion

The aim of the present study was to compare mean REEs estimated by specific formulas recently developed for CKD patients with REE values measured by IC, the gold standard method for this measurement. We found that the formula developed by Fernandes et al. (2021) [15] had the lowest mean bias, the best correlation, and the best accuracy for patients in our study. The superiority of the Fernandes et al. 2021 formula over other formulas may be partly due to the fact that it includes GFR and DM variables in addition to demographic/anthropometric characteristics. Thus, it seems to be a more realistic formula for non-dialytic CKD patients [21,22].

Nutritional balance in CKD is essential to avoid protein-energy wasting or obesity, conditions strongly associated with adverse outcomes such as disease progression, dialysis initiation, metabolic syndrome, and mortality [1,2]. Obtaining reliable REE values using predictive formulas is essential to better adjust individual energy and protein intakes [8,23].

Previous predictive formulas for REE, such as those of Harris and Benedict (1919) [9] and Schofield (1985) [10], have been studied and shown to be inconsistent with values of REE measured by IC in the CKD population. Kamimura et al. (2011) [7] tested the REE prediction accuracies of the Harris–Benedict and Schofield formulas in non-dialytic CKD patients treated by hemodialysis and peritoneal dialysis. Our group also previously tested the agreement of the Harris–Benedict formula in pre-dialysis and dialysis populations [12]. In all three studies, the estimated REE values were overestimated.

With the emerging need for specific CKD REE formulas, Vilar et al. [11] developed a prediction equation using only anthropometric measures such as the height, weight, sex, and age of hemodialysis patients. Byham-Gray et al. [13] included glycosylated hemoglobin levels in addition to these anthropometric variables and developed two equations, one with serum creatine and another with serum protein C-reactive levels, differentiated by sex. Both formulas were validated for the population undergoing hemodialysis and can be reliably used in this population.

In 2019, Fernandes et al. [14] used the variables of age, body weight, and fat-free mass to develop two formulas, differentiated for men and women, for hemodialysis and peritoneal dialysis. In our comparative analysis, limits of agreement were high (−42.96 to 484.63 kcal) with these formulas, which can generate discrepant values, both underestimating and overestimating real REE values. Similar results were observed when comparing Byham-Gray formulas [13]; the formula using the CRP value came close to the average bias generated by IC, but it also showed high limits of agreement (−628.90 to 384.30 kcal), as did the formula using the serum creatinine value (−587.76 to 346.34 kcal); in graphs of these specific formulas presented herein, the size of the spheres indicates the greater variation in results with repeated measurements than in the other formulas. The equation of Vilar et al. [11] produced significantly higher REE values than those measured by IC, indicating an overestimation of the true REE values of our population, and showed high mean biases.

Bailey and Cols [23] validated the use of the Vilar [11], Fernandes [14,15], and Byham-Gray [13] equations for the dialysis population, a secondary analysis of data previously collected from the Rutgers Nutrition and Kidney Database (RNKD) [23]. These specific equations had varying degrees of accuracy; therefore, the authors emphasized the need to understand these variations and correct the imprecision of the formulas for use in clinical practice; for this reason, most formulas have relatively many parameters, which can be a problem when used in routine practice. Despite this complication, formulas with parameters that characterize specific diseases can produce more reliable and reproducible results.

Of the three equations developed by these authors, the one using data on body weight, age, eGFR according to sex, and the presence of diabetes [14] produced the most similar results to the values of IC in the present study, as evidenced by the significantly low mean bias (0.15 [−653.51; 398.90]) and better correlation (r = 0.789; *p* = 0.000), although it also presented a wide range of acceptable agreement (−594.37 to −446.92 kcal). Although still with high limits of agreement, it was also the formula with the highest accuracy, at ±20%.

The accuracy of ±10% (90–110% of REE using IC) [24] is used to validate or not the use of formulas in clinical practice. Using data from this study as an example, it could be argued that 10% of the REE value measured by IC (~134 kcal) may not be that relevant for caloric adjustment. Analyzing the accuracy at ±20% (i.e., ~268 kcal) would lead to more impactful differences in clinical practice when discussing caloric support in the nutritional management of patients.

This study had several limitations. The number of patients who completed the three assessments and were included was small. In addition, the patients included had certain baseline characteristics that may have selected the sample and biased the results. Despite its limitations, this study has some distinctive points: the statistical methodology used is sophisticated and not often used in the literature. In addition, finding a predictive formula with good accuracy makes it possible to optimize nutritional management and reduce the risk of poor nutritional outcomes in patients with CKD.

## 5. Conclusion

The prediction formula for REE that showed the least bias, best correlation, and best accuracy of ±20% compared to REE measured by IC was the formula developed by Fernandes et al. (2021) [15].

## Figures and Tables

**Table 1:** Prediction formulas’ details.

Authors	Formulas	Reference
Fernandes et al., 2019 Country: Brazil	REE (kcal/day) = 956.02 − (8.08 × age) + (11.07 × body weight (Kg)) + 136.4 (if man)	[14]
Fernandes et al., 2021 Country: Brazil	REE (kcal/day) = 854 + (7.4 × body weight (Kg)) + (179 × sex) − (3.3 × age) + (2.1 × eGFR) + 26 (if DM) In: sex: male = 1; female = 2; DM = diabetes mellitus	[15]
Byham-Gray et al., 2017 (MHDE-CRP) Country: USA	REE male (Kcal/day) = 1027.8 − (5.19 × age) + (9.67 × body weight) + (2.71 × CRP) REE female (Kcal/day) = 820.47 − (5.19 × age) + (9.67 × body weight) + (2.71 × CRP) In: Body weight in kilograms (after dialysis) and CRP in mg/dL. If CRP is not available, A1C or serum creatinine can replace this model.	[13]
Byham-Gray et al., 2017 (MHDE-SCr) Country: USA	REE male (Kcal/day) = 1024.41 − (4.9 × age) + (10.21 × body weight) − (3.25 × serum creatinine) REE female (Kcal/day) = 802.0 − (4.9 × age) + (10.21 × body weight) − (3.25 × serum creatinine) In: Body weight in kilograms (after dialysis) and serum creatinine in mg/dL.	[13]
Villar et al., 2014 Country: United Kingdom	REE (Kcal/day) = −2.497 × Factor_age_ + (0.011 × height (cm) × 2.023 × weight (Kg) × 0.6291) + 68.171 × Factor_sex_ In: Factor_age_ = 1 if 65 years or older and 0 if younger than 65, and Factor_sex_ = 1 if male and 0 if female.	[11]

REE: resting energy expenditure; eGFR: estimated glomerular filtration rate; DM: diabetes mellitus; CRP: c-reative protein.

**Table 2:** Demographic and clinical variables (*n* = 53, IC measures = 159).

Variables	Patients
Demographic parameters	
Mean age (years)	65 ± 13
Black race (%)	10 (19)
Female (%)	20 (38)
Clinical parameters	
Causes of chronic kidney disease	
Diabetes mellitus (%)	20 (37.7)
Hypertension (%)	20 (37.7)
Glomerulonephritis (%)	12 (22.4)
eGFR (mL/min/1.73 m^2^)	12.43 ± 3.65
CPR (mg/dL)	1.55 (0.5–6.5)
REE by IC mean (Kcal/day)	1341.38 ± 371.05
RQ mean	0.99 ± 0.19
Nutritional parameters	
Body weight (Kg)	74.72 ± 14.36
Body mass index (Kg/m^2^)	27.84 ± 4.56

eGFR: estimated glomerular filtration rate; CPR: C-reative protein; REE: resting energy expenditure; IC: indirect calorimetry; RQ: respiratory quotient.

**Table 3:** Comparison between REEs measured by IC and predictive formulas.

	Mean ± SD	*p*	Bland–Altman Analysis		Lin’s Correlation Coefficient	Accuracy ± 10%	Accuracy ± 20%
			Bias Mean	Agreement Limits			
**Indirect calorimetry**	**1341.4 ± 371.0**						
[14]	1261.6 ± 218.3	<0.000	90.70	−547.45 to −393.80	0.5731	31.3%	78.2%
[15]	1367.4 ± 367.5	<0.000	15.02	−653.51 to −398.90	0.7892	32.4%	85.1%
[13]	1309.3 ± 226.0	<0.000	17.23	−581.90 to −437.64	0.6520	41.9%	75.4%
[13]	1352.1 ± 242.8	<0.000	−21.62	−620.97 to −476.25	0.6672	46.1%	78.8%
[11]	1494.5 ± 231.7	<0.000	−142.25	−793.22 to −636.48	0.5283	38.4%	64.6%

## Data Availability

The data presented in this study are available on request from the corresponding author. The data are not yet publicly available due to future publication in placement.
